# 

**Published:** 1961-04

**Authors:** 


					I 6^
^ zr?
THE
MEDICAL JOURNAL
OF THE
SOUTH-WEST
Incorporating
The Bristol Mcdico-Chirurgical Jour
April 1961
?L- 76 (ii). No. 280 Price 4/-
V \ I

				

